# A Reference Hand-Book of the Medical Sciences

**Published:** 1889-12

**Authors:** 


**A Reference Hand-Book of the Medical Sciences,** embracing the entire range of Scientific and Practical Medicine and Allied Science. By various writers. Illustrated by chromo-lithographs and fine wood engravings. Edited by ALFRED H. BUCK, M. D. Volume VIII. Containing an appendix (523 pp.) and a general index (197 3-column pp.) New York: William Wood & Co.

This volume of the Reference Handbook of the Medical Sciences completes this extensive and valuable work. In examining the eight volumes, of which the series is composed, we are frank to state that, in the variety of subjects selected and in the ability of the writers, the handbook presents a compendium which will constitute an excellent library for the general practitioner, and an extensive reference book for the student. Great credit is due to the accomplished editor for the able manner in which he has performed his duty, and the zeal and enterprise of the publishers are demonstrated in the paper and typography, and also in engravings, etc., with which the volumes are embellished.

The eighth volume is, for the most part, taken up with the appendix and index. The appendix has enabled the editor to supplement the subjects, arranged in alphabetical order, with important and valuable monographs, which makes the work all the more complete.

For the purpose named in the title, we regard this work one of the most important issued by Messrs. Wood & Co. It is just the series for the practitioner, affording him, in a condensed form, much instructive matter which, in the ordinary medical library, he does not possess.

